# Dry Eye Disease and Tear Cytokine Levels—A Meta-Analysis

**DOI:** 10.3390/ijms21093111

**Published:** 2020-04-28

**Authors:** Matilde Roda, Ivan Corazza, Maria Letizia Bacchi Reggiani, Marco Pellegrini, Leonardo Taroni, Giuseppe Giannaccare, Piera Versura

**Affiliations:** 1 Ophthalmology Unit, DIMES, Alma Mater Studiorum University of Bologna and S.Orsola-Malpighi Teaching Hospital, 40138 Bologna, Italy; matilde.roda@hotmail.com (M.R.); marco.pellegrini@hotmail.it (M.P.); leonardo.taroni1@gmail.com (L.T.); 2 Department of Experimental, Diagnostic and Specialty Medicine, S. Orsola-Malpighi University Hospital, 40138 Bologna, Italy; ivan.corazza@unibo.it (I.C.); maria.bacchireggiani@unibo.it (M.L.B.R.); 3 Department of Ophthalmology, University Magna Graecia of Catanzaro, 88100 Catanzaro, Italy; giuseppe.giannaccare@gmail.com

**Keywords:** tear cytokines, tear chemokines, dry eye, meta-analysis

## Abstract

Background—It is recognized that inflammation is an underlying cause of dry eye disease (DED), with cytokine release involved. We systematically reviewed literature with meta-analyses to quantitatively summarize the levels of tear cytokines in DED. Methods—The PubMed, Embase, Web of Science, Ovid, Cochrane, and Scopus databases were reviewed until September 2019, and original articles investigating tear cytokines in DED patients were included. Differences of cytokines levels of DED patients and controls were summarized by standardized mean differences (SMD) using a random effects model. Study quality was assessed by applying Newcastle-Ottawa-Scale and the GRADE quality score. Methods of analytical procedures were included as covariate. Results—Thirteen articles investigating 342 DED patients and 205 healthy controls were included in the meta-analysis. The overall methodological quality of these studies was moderate. Systematic review of the selected articles revealed that DED patients had higher tear levels of interleukin (IL)-1β, IL-6, chemokine IL-8, IL-10, interferon-γ, IFN-γ, and tumor necrosis factor-α, TNF-α as compared to controls. Evidence was less strong for IL-2 and IL-17A. Conclusions—Data show that levels of tear cytokines in DED and control display a great variability, and further studies of higher quality enrolling a higher number of subjects are needed, to define a cut-off value.

## 1. Introduction

Dry eye disease (DED) affects millions of patients worldwide with a prevalence ranging from 5 to 50% and increasing with age [1], and is one of the most frequent causes of visits in the ophthalmic daily practice [2]. Visual disturbances and subjective discomfort symptoms or pain can significantly impact patient’s quality of life [3]. The definition of DED given in the TFOS DEWS (Tear Film & Ocular Surface Society) II consensus [4] includes inflammation as one of the key elements contributing to the onset and triggering a self-sustaining vicious circle.

Recent studies have suggested that pro-inflammatory cytokines in tears exert a key role in the pathogenesis of several ocular surface diseases, including DED [4,5], and Pflugfelder and co-workers demonstrated increased levels of IL-1, IL-6, and IL-8 in Sjogren’s syndrome (SS) patients, and these increased concentrations were associated with the severity of DED clinical parameters, such as greater corneal staining and lower tear secretion [6,7,8].

The search for a marker of inflammation in the tears generated a great deal of research and several papers on the levels of tear cytokines in patients with DED of different severity, before and after several therapeutic approaches, have been published. However, despite the large body of evidence of the role of cytokine in the vicious circle of DED [4], no consensus has been reached so far as regards tear collection, methods of analysis, cut-off values, and panel of cytokines more involved in ocular surface disease. A systematic review with a quantitative synthesis of the cytokine profiles of DED patients compared to controls has not been performed until now. The aim of this meta-analysis was therefore to systematically and quantitatively review data on pro-inflammatory cytokines and chemokines in DED patients and controls.

## 2. Results

### 2.1. Characteristics of the Included Studies

**Figure 1:**
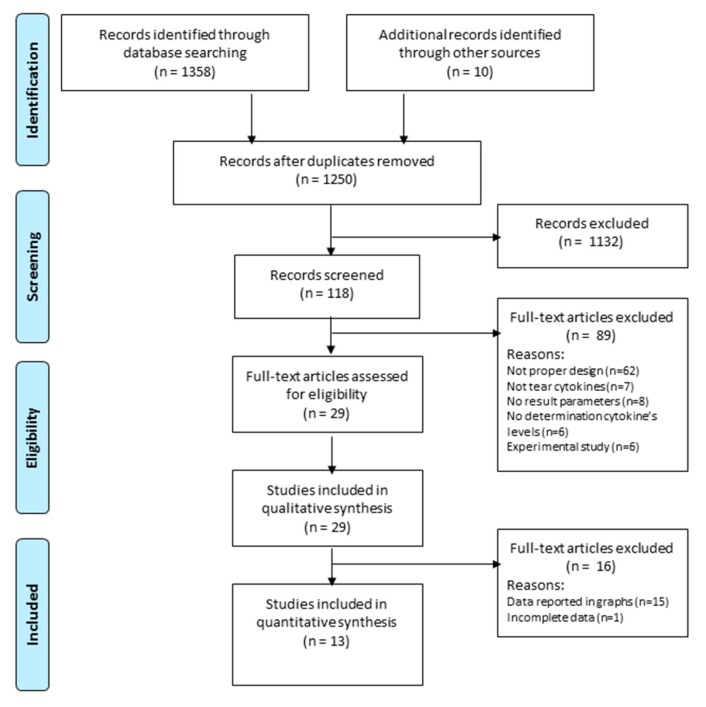
Flow chart PRISMA (Preferred Reporting Items for Systematic Reviews and Meta-Analyses).

Based on the search strategy, 1368 studies were retrieved from the relevant databases, reduced to 1250 records after duplicates removed. Of these, 1132 were excluded after our primary screening as not meeting the inclusion criteria. In addition, we recorded all reasons for study exclusion, disagreements between the two reviewers and third reviewer comments. Of the 118 articles screened, 29 were included for the qualitative synthesis and only 13 were finally included in the meta-analysis (Figure 1) [7,8,9,10,11,12,13,14,15,16,17,18,19]. In these thirteen studies the most frequent analyzed cytokines were: IL-1β in eight studies [7,8,9,10,16,17,18,19], IL-2 in five studies [9,11,13,16,19], IL-6 in eleven studies [7,9,11,12,13,14,15,16,17,18,19], IL-8 in six studies [7,9,13,16,17,19], IL-10 in six studies [9,11,13,16,17,19], IL-17A in five studies [9,10,11,12,13], TNF-α in eight studies [9,11,12,13,15,16,17,19], and IFN-γ in four studies [9,13,17,19].

### 2.2. Study and Patient Characteristics.

Studies were published between 1998 and 2019. The quality score of the included studies varied from 4 to 9 with mean score of 7 stars (Table 1). According to the grade quality analysis, all the included studies were graded as moderate value of evidence i.e., grade B. A summary of the included studies with the analyzed cytokines is given in Table 2.

The ELISA technique was used for detection of cytokines in five out of thirteen included studies [8,12,14,15,18], the MULTIPLEX techniques in other seven studies [7,9,10,13,16,17,19] and the BDTM cytometric bead array in one study [11].

A total of 342 DED patients and 205 healthy non DED controls, from the thirteen studies in the quantitative analysis, were included. In all studies, patients with DED and healthy controls were matched for age, but not for sex. Information regarding the number of male and female subjects was available in most of the included studies, and there was a total of 220 (40.2%) males (140 patients with DED and 80 controls) and 327 (59.8%) female participants (202 patients with DED and 125 controls). The mean age of DED patients was 52.2 years (ranged from 33.5 to 69.7 years) compared to 42.6 years (ranged from 32.2 to 59.1 years) in control subjects.

An I^2^ index >50% was found in all meta-analyses, consequently forest plots reporting only the random effect method were graphed. 

### 2.3. IL-1β

**Figure 2:**
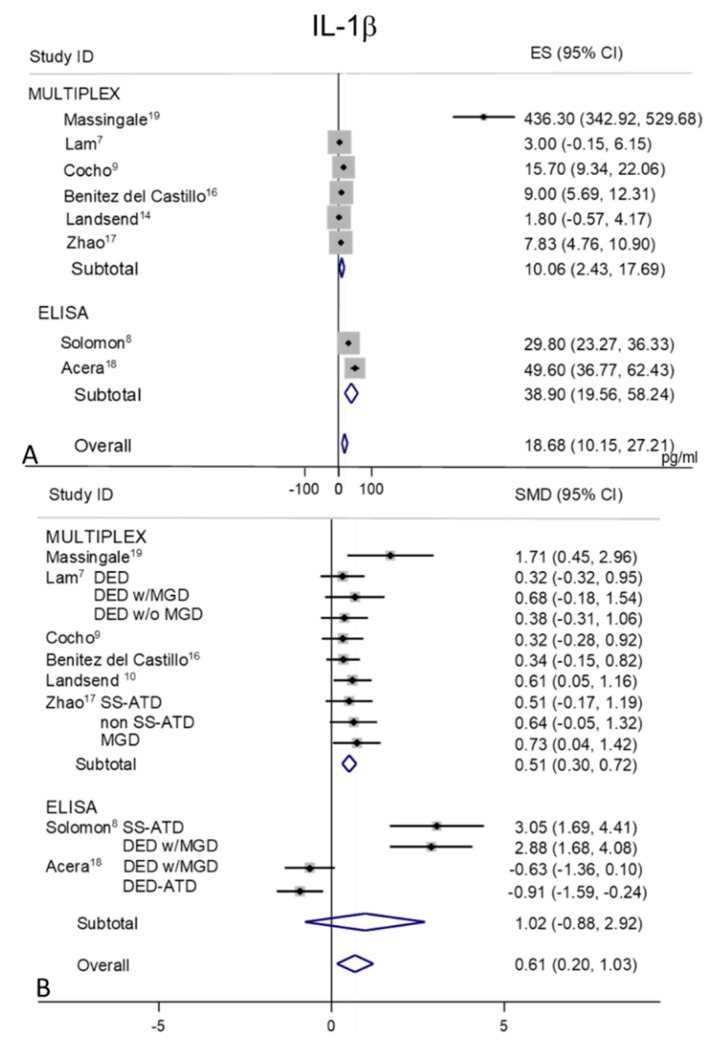
Meta-analysis of tear IL-1β levels: (**A**) mean values and 95% CI of control subjects; (**B**) standardized mean difference (SMD) of DED patients vs. control subjects. ES = effect size. DED = dry eye disease; SS = Sjogren’s syndrome; non SS = non Sjogren’s syndrome; ATD = aqueous tear deficiency; MGD = meibomian gland dysfunction.

Tear levels of IL-1β were extracted from eight studies, MULTIPLEX techniques of analysis were used in six of these studies [7,9,10,16,17,19] and ELISAs were used in the remaining two [8,18] (Figure 2A,B). In control subjects (Figure 2A), a considerable heterogeneity was observed in the MULTIPLEX sub-group (I^2^ = 95.5%, *p* = 0.0001) and in the ELISA subgroup group (I^2^ = 86.2%, *p* = 0.007) with an overall I^2^ = 96.6% (*p* = 0.0001). The pooled mean value for IL-1β in controls appeared as 18.68 pg/mL (95% CI 10.15–27.21 pg/mL; *p* = 0.0001).

The pooled mean valued for DED was 31.54 pg/mL (95% CI 23.93–39.15 pg/mL, *p* = 0.0001) with an overall I^2^ = 98.4% (*p* = 0.0001).

Random-effects meta-analysis demonstrated a significance for higher tear concentrations in patients with DED (*n* = 254) as compared to non DED controls (*n* = 147) (overall pooled SMD 0.61; 95% CI 0.20 to 1.03; *p* = 0.004) (Figure 2B). In studies performed with the MULTIPLEX techniques the difference between DED and controls mean values were lower than their variability, whereas in the studies performed with the ELISA techniques the difference in means between DED and controls mean values were greater than their variability. These differences might be due to the contribution of a specific paper published over ten years ago [19], and could be linked to technical issues of the methods/kits used for cytokine measurements, which could have been changed or optimized more recently. However, by also removing this paper from the analysis, the heterogeneity remained high (I^2^ = 95.1%, *p* = 0.0001).

### 2.4. IL-2

**Figure 3:**
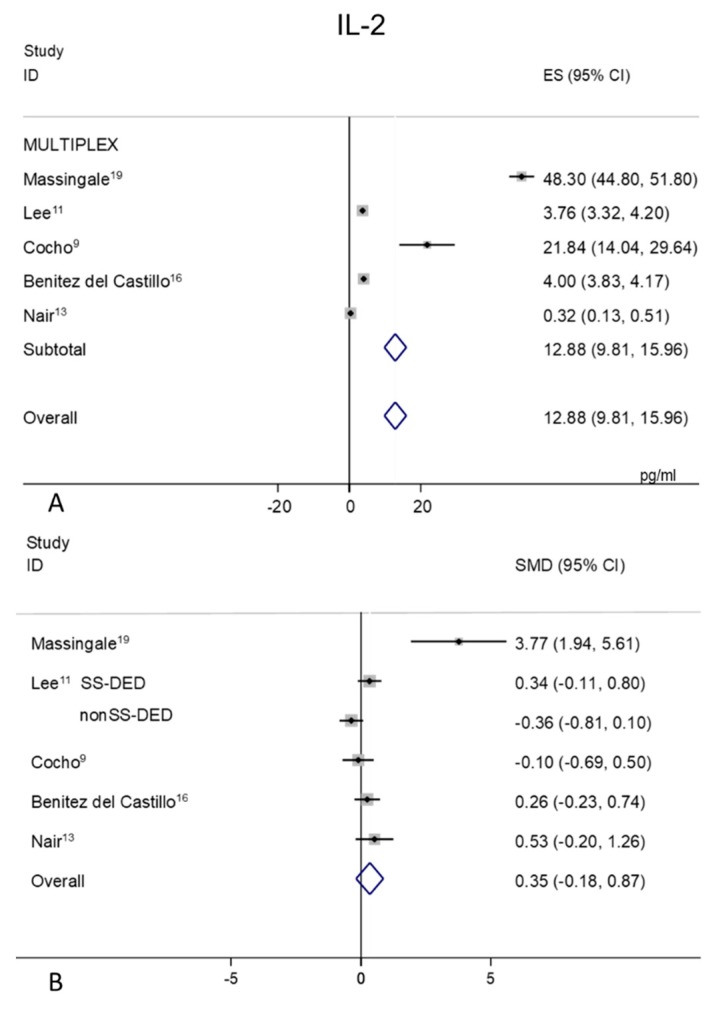
Meta-analysis of tear IL-2 levels: (**A**) mean values and 95% CI of control subjects; (**B**) standardized mean difference (SMD) of DED patients vs. control subjects. ES = effect size; DED = dry eye disease; SS = Sjogren’s syndrome; non SS = non Sjogren’s syndrome.

Tear levels of IL-2 were extracted from five studies all performed with the MULTIPLEX analysis [9,11,13,16,19] (Figure 3A,B). Random-effects meta-analysis demonstrated significant tear concentrations in patients with DED (*n* = 159) as compared to non DED controls (*n* = 111). A great heterogeneity was observed in both controls (99.7%, *p* = 0.0001) (Figure 3A) and DED patients (96.7%, *p* = 0.0001). The overall mean IL-2 concentration was 12.88 pg/mL (95% CI 9.81–15.96 pg/mL, *p* = 0.0001) versus 6.84 pg/mL in DED patients (95% CI 4.00–9.69 pg/mL, *p* = 0.0001). Due to the SMD heterogeneity among studies (I^2^ = 77.5%, *p* = 0.0001) and small differences in mean values of DED and control patients, elusive conclusion about IL-2 values trend can be assumed (Figure 3B, *p* = 0.197).

### 2.5. IL-6

**Figure 4:**
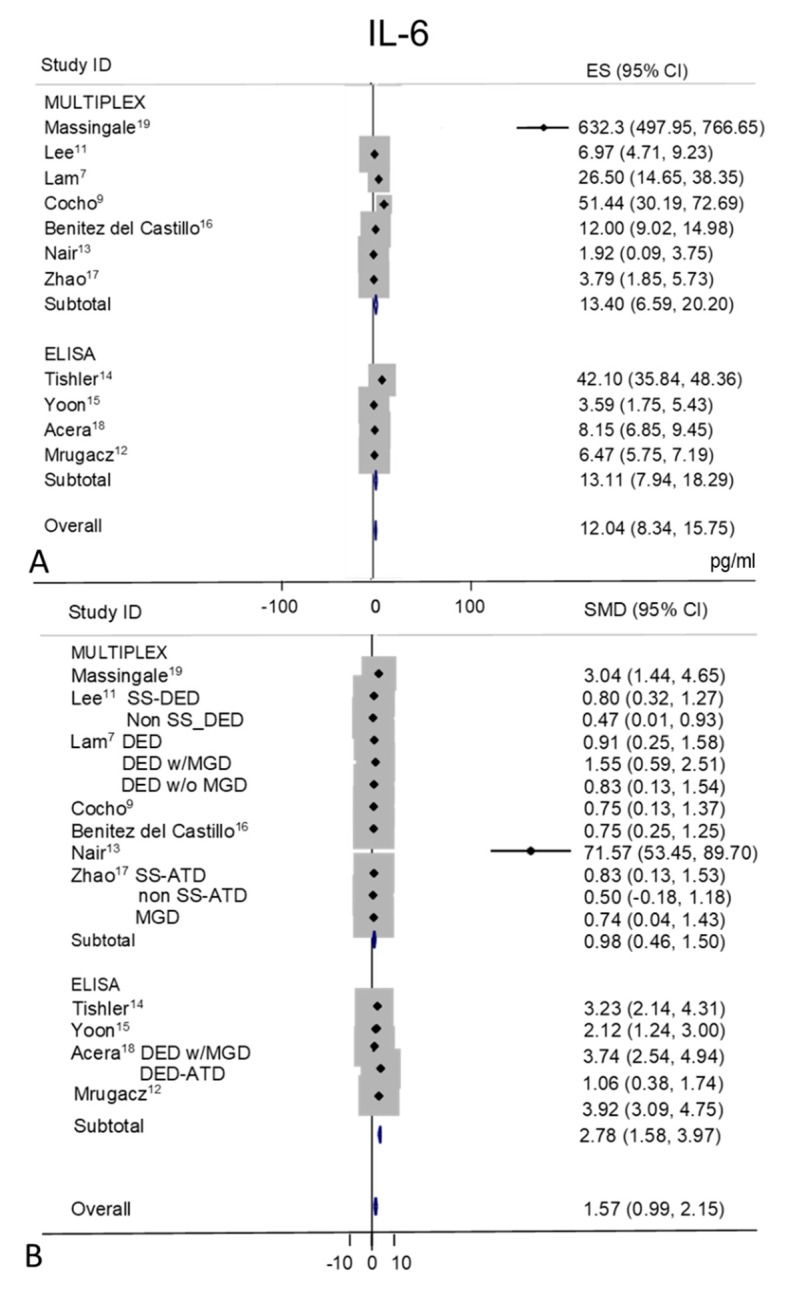
Meta-analysis of tear IL-6 levels: (**A**) mean values and 95% CI of control subjects; (**B**) standardized mean difference (SMD) of DED patients vs. control subjects. ES = effect size. DED = dry eye disease; SS = Sjogren’s syndrome; non SS = non Sjogren’s syndrome; ATD = aqueous tear deficiency; MGD = meibomian gland dysfunction.

Tear IL-6 levels were extracted from eleven studies, MULTIPLEX techniques of analysis were used in seven of these studies [7,9,11,13,16,17,19] and ELISA in the remaining four [12,14,15,18] (Figure 4A,B). Random-effects meta-analysis demonstrated a significance for higher tear concentrations in patients with DED (*n* = 337) as compared to non DED controls (*n* = 258). IL-6 level was significantly increased in tears of patients with DED in all studies, except in the study by Cocho et al. [9] (*p* = 0.11). A great heterogeneity was observed in control values (I^2^ = 96.6%, *p* = 0.0001) with an overall estimation of 12.04 pg/mL (95% CI 8.34–15.75, *p* = 0.0001) and of 98.82 pg/mL (95% CI 46.75–150.89 pg/mL, *p* = 0.0001) in DED group. Tear IL-6 mean values were greater in DED as compared to control mean values in the ELISA studies with a pooled SMD of 2.78 pg/mL (95% CI 1.58–3.97, *p* = 0.0001) while in the MULTIPLEX studies the pooled difference was 0.98 pg/mL (95% CI 0.46–1.50, *p* = 0.0001), with an overall effect of 1.57 pg/mL (95% CI 0.99–2.15 pg/mL; *p* = 0.0001).

### 2.6. IL-8

**Figure 5:**
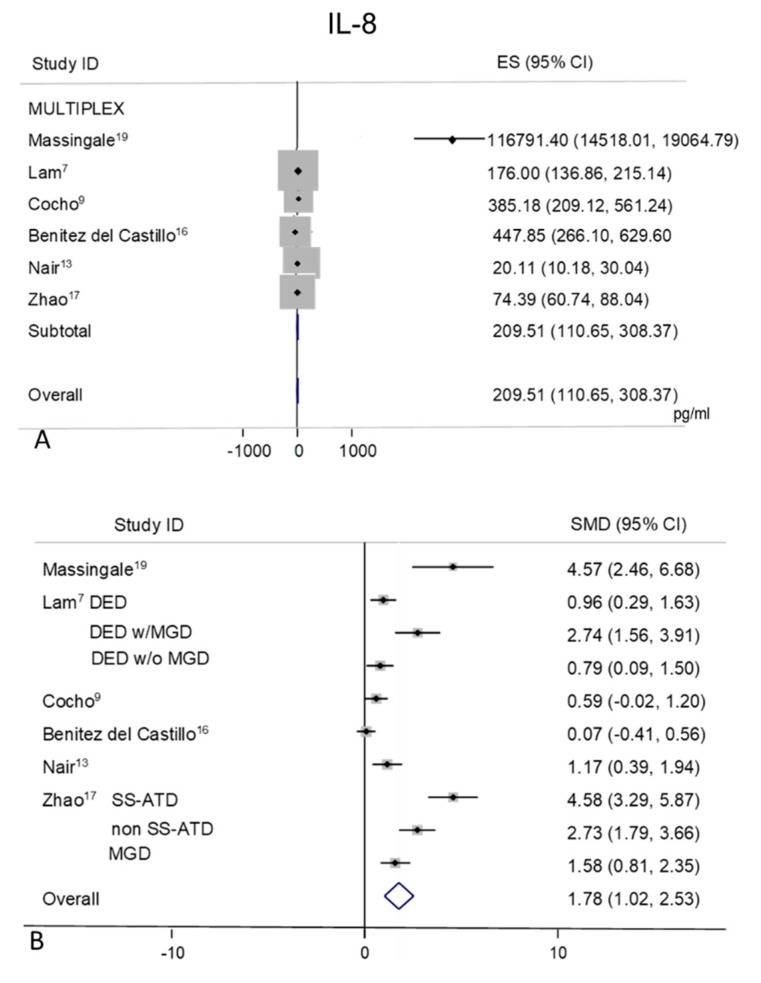
Meta-analysis of tear IL-8 levels: (**A**) mean values and 95% CI of control subjects; (**B**) standardized mean difference (SMD) of DED patients vs. control subjects. ES = effect size; DED = dry eye disease; SS = Sjogren’s syndrome; non SS = non Sjogren’s syndrome; ATD = aqueous tear deficiency; MGD = meibomian gland dysfunction.

Tear levels of IL-8 were extracted from six studies [7,9,13,16,17,19] (Figure 5A,B), all performed with MULTIPLEX based methods. The studies showed a great heterogeneity of mean values both in controls (I^2^ = 98.5%, *p* = 0.0001) with an overall pooled mean value of 209.51 pg/mL (95% CI 110.65–308.37 pg/mL, *p* = 0.0001) and in DED group (I^2^ = 97.0%, *p* = 0.0001) with an overall mean value of 501.48 pg/mL (95% CI 319.67–683.30 pg/mL, *p* = 0.0001).

Random-effect meta-analysis demonstrated a significant difference in patients with DED (*n* = 261) as compared to non DED controls (*n* = 128) (pooled SMD 1.78; 95% CI 1.02–2.53, *p* = 0.0001).

### 2.7. IL-10

**Figure 6:**
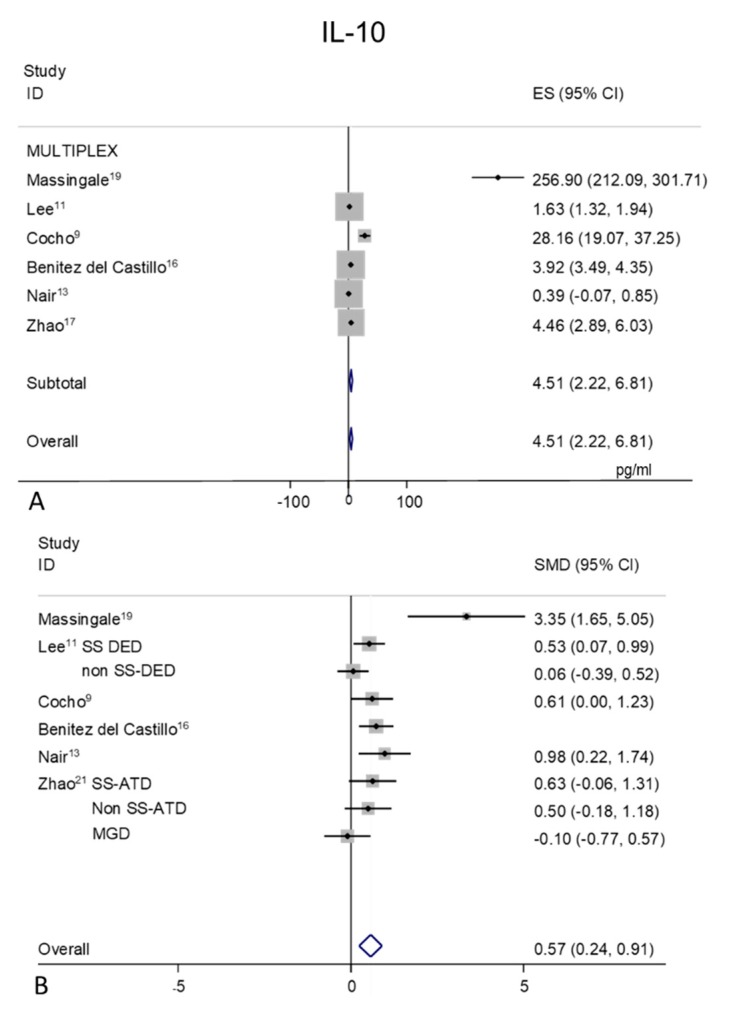
Meta-analysis of tear IL-10 levels: (**A**) mean values and 95% CI of control subjects; (**B**) standardized mean difference (SMD) of DED patients vs. control subjects. ES = effect size. DED = dry eye disease; SS = Sjogren’s syndrome; non SS = non Sjogren’s syndrome; ATD = aqueous tear deficiency; MGD = meibomian gland dysfunction.

Tear levels of IL-10 were extracted from six studies [9,11,13,16,17,19], all detected with MULTIPLEX analysis (Figure 6A,B). A great heterogeneity was observed (I^2^ = 98.3%, *p* = 0.0001) in the mean values of the control subjects (Figure 6A), with an overall pooled mean value of 4.51 pg/mL (95% CI 2.22–6.81 pg/mL, *p* = 0.0001). A great heterogeneity (I^2^ = 97.4%, *p* = 0.0001) is observed in the DED group (mean pooled value 7.87 pg/mL, CI 95% 3.23–12.52 pg/mL, *p* = 0.001), too.

Random-effect meta-analysis showed a trend toward significance for higher tear concentrations in patients with DED (*n* = 264) as compared to non DED controls (*n* = 180), with an overall estimation of 0.57 (95% CI 0.24–0.91, *p* = 0.001).

### 2.8. IL-17A

**Figure 7:**
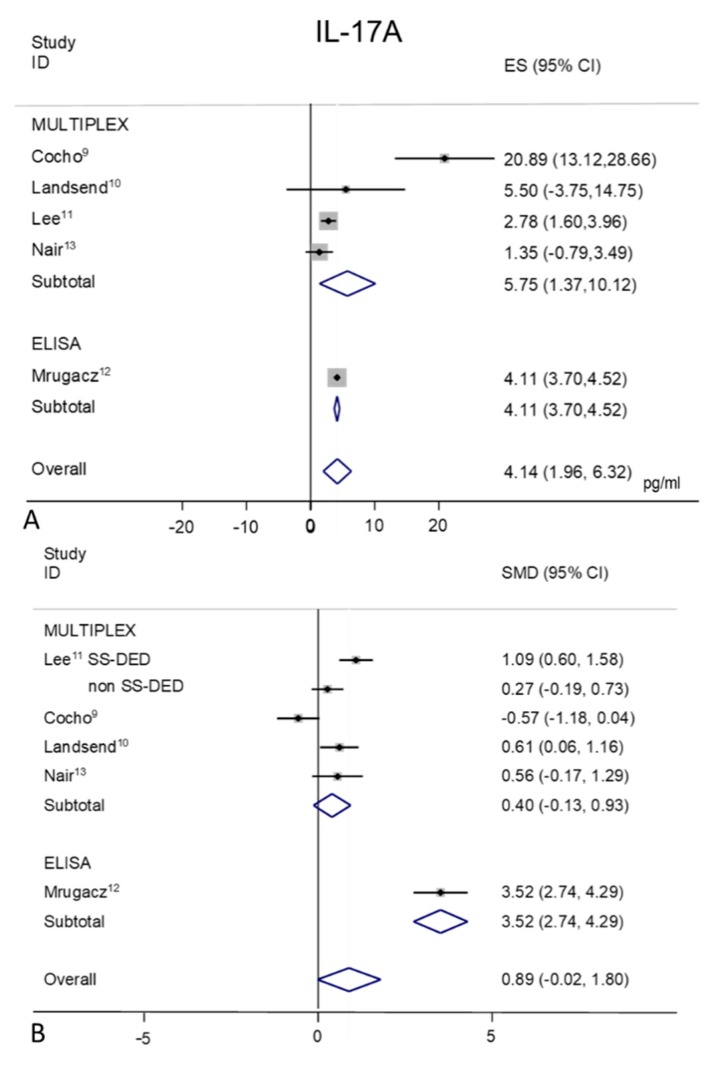
Meta-analysis of tear IL-17A levels: (**A**) mean values and 95% CI of control subjects; (**B**) standardized mean difference (SMD) of DED patients vs. control subjects. ES = effect size; DED = dry eye disease; SS = Sjogren’s syndrome; non SS = non Sjogren’s syndrome.

Tear IL-17A concentration were extracted from four Multiplex used studies [9,10,11,13] and 1 ELISA used study [12] (Figure 7A,B). Random-effect meta-analysis demonstrated a trend toward significance for higher tear concentrations in patients with DED (*n* = 189) as compared to non DED controls (*n* = 158). Tear IL-17A levels were significantly greater in various ocular surface inflammatory diseases (including SS, not SS-DED, MGD, aniridia and oGVHD) as compared to control subjects, supporting a role for IL-17A in the immune pathogenesis of DED. Only in one study [9] there wasn’t any statistically significant difference between oGVHD-DED group and healthy controls (*p* = 0.19).

In the MULTIPLEX group, the heterogeneity (I^2^ = 86.9%, *p* = 0.0001) in the control mean values is mainly due to the work by Cocho et al. [9] (Figure 7A), that leads to inaccurate overall estimation of 4.14 pg/mL (95% CI 1.96–6.32 pg/mL; *p* = 0.0001).

A heterogeneity of 96.9% (*p* = 0.0001) is observed in DED group, with an overall pool estimation of 7.87 pg/mL (95% CI 3.23–12.52 pg/mL, *p* = 0.001).

The forest plot of IL-17A presents different standardized mean differences inside the two sub-groups (Figure 7B); in the MULTIPLEX sub group (SMD 0.40, 95% CI −0.13–0.93; *p* = 0.140) and larger differences in the ELISA study group (SMD 3.52; 95% CI 2.74–4.29 *p* = 0.0001). The overall SMD results as 0.89 (95% CI −0.02–1.80; *p* = 0.054)

### 2.9. TNF-α

**Figure 8:**
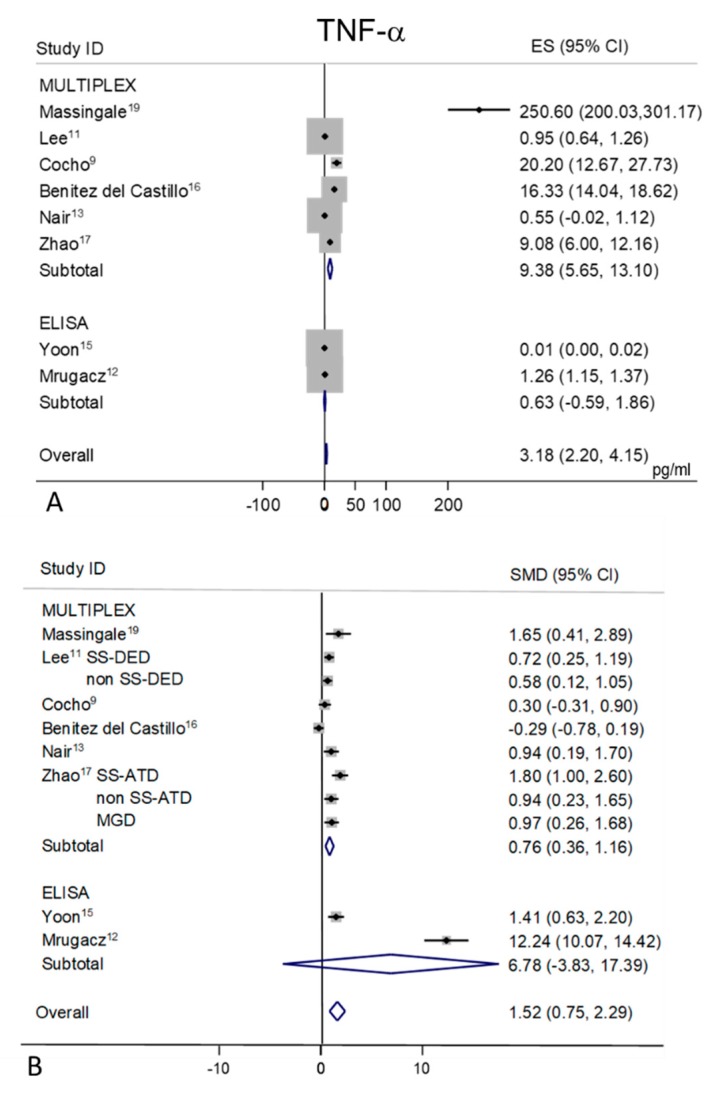
Meta-analysis of tear TNF-α levels: (**A**) mean values and 95% CI of control subjects; (**B**) standardized mean difference (SMD) of DED patients vs. control subjects. ES = effect size; DED = dry eye disease; SS = Sjogren’s syndrome; non SS = non Sjogren’s syndrome; ATD = aqueous tear deficiency; MGD = meibomian gland dysfunction.

Tear levels of TNF-α were extracted from eight studies, six detected with the MULTIPLEX analysis [9,11,13,16,17,19], and two with the ELISA [12,15] (Figure 8A,B). Random-effects meta-analysis demonstrated a trend toward significance for higher tear concentrations in patients with DED (*n* = 244) as compared to non DED controls (*n* = 214), only for the MULTIPLEX subgroup (SMD 0.76; 95% CI 0.36–1.16; *p* = 0.0001). The ELISA subgroup showed no statistically significant difference between DED and controls (*p* = 0.21). A considerable heterogeneity in controls values in each sub-group (I^2^ = 98.4% and 99.8% respectively, *p* = 0.0001 for both) was found (Figure 8A), with a mean pool value of 3.18 pg/mL (95% CI 2.20–4.15 *p* = 0.0001). The mean pool value for the DED group resulted in 12.16 pg/mL (95% CI 6.92–17.40 pg/mL, *p* = 0.0001) with an overall heterogeneity of 99.3% (*p* = 0.0001). The overall pool SMD (1.52; 95% CI 0.75–2.29, *p* = 0.0001) is influenced in particular by the heterogeneity of one ELISA study [12], (Figure 8B).

### 2.10. IFN-γ

**Figure 9:**
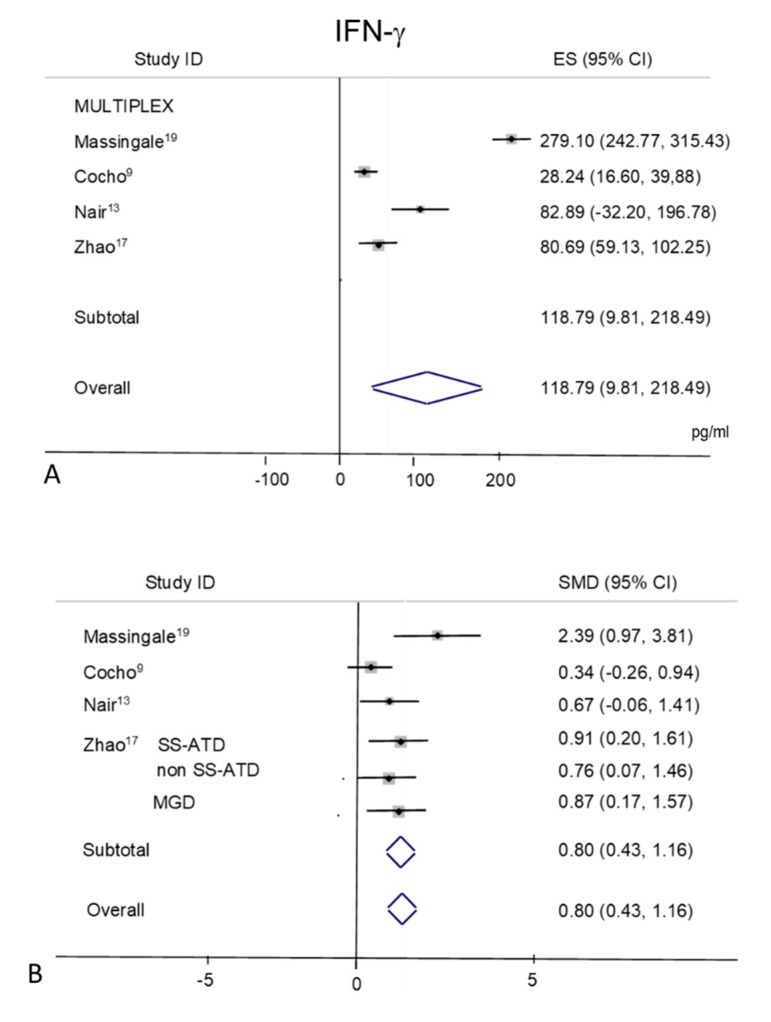
Meta-analysis of tear IFN-γ levels: (**A**) mean values and 95% CI of control subjects; (**B**) standardized mean difference (SMD) of DED patients vs. control subjects. ES = effect size; DED = dry eye disease; SS = Sjogren’s syndrome; non SS = non Sjogren’s syndrome; ATD = aqueous tear deficiency; MGD = meibomian gland dysfunction.

Tear levels of IFN-γ were extracted from four studies [9,13,18,19] all performed with the MULTIPLEX based techniques (Figure 9A,B). In control subjects (Figure 9A), a considerable heterogeneity was observed among the studies with an overall I^2^ = 98.3% (*p* = 0.0001). The pooled mean value for IFN-γ in controls appeared as 118.79 pg/mL (95% CI, 19.10–218.49 pg/mL, *p* = 0.02) (Figure 9A). A high heterogeneity is also present among DED values (I^2^ = 97.2%, *p* = 0.0001), with a mean pool value of 183.50 pg/mL (95% CI 104.18–262.82 pg/mL, *p* = 0.0001). Random-effects meta-analysis demonstrated a significance for higher tear concentrations in patients with DED (*n* = 174) as compared to non DED controls (*n* = 95) (overall pooled SMD 0.80; 95% CI, 0.43–1.17; *p* = 0.0001) (Figure 9B).

In the Supplementary Material, a table (Table S1) is provided, summarizing all the values obtained from meta-analysis for each cytokine and each group (controls and DED).

## 3. Discussion

This meta-analysis reports significantly higher concentrations of the tear inflammatory mediators IL-1β, IL-6, IL-8, IL-10, IFN-γ, and TNF-α, in the tears of DED patients as compared to age-matched non DED control subjects. Conversely, the evidence of difference was less strong for IL-17A and IL-2. Most studies reported large standard deviations, suggesting substantial inter-individual variation in cytokine concentrations that was not explained. Overall, these meta-analytic results strengthen the evidence that DED is accompanied by release of cytokines in tears, suggesting a panel indicative of the inflammatory response, in agreement with previous reports [20,21]. However, the analysis of tear cytokines as a biomarker for DED can still be considered an unmet need, as a great variability in the procedures of tear collection, storage, and analytical methods might have generated inconsistent data, not allowing the estimation of either a reference interval for control subjects and a cut-off value between the DED and controls. For instance, different sampling procedures may determine different concentrations of cytokines detected, as it has been shown in previous papers analyzing the impact of different procedures, including basal tears, flush tears, tear samples recovered from Schirmer test strips or sponges [22,23,24]. In addition, there are few published manuscripts presented a validated and standard operative procedure (SOP) to measure tear cytokines [25,26,27]. One limitation of our analysis is to have grouped as DED either aqueous deficient dry eye (ADDE), evaporative dry eye (EDE), and meibomian gland dysfunction (MGD), and not having stratified patients based on etiology with the aim to examine them separately. However, according to the recent TFOS DEWS II report [4], it is recognized that the two predominant etiologies of ADDE and EDE are often overlapping, and MGD, a major contributor to EDE, is considered the leading cause of dry eye in clinic and population-based studies. The severity of DED was also not considered into the analysis even though it is associated with the tear cytokine concentrations. However, it is not possible to stratify for analysis based on the severity due to the lack of power.

Although tear cytokines have been investigated in a large number of studies, in addition to clinical confounders such as disease severity and not homogeneous criteria for DED diagnosis, variability in assay procedures may have contributed substantially to heterogeneity. Conventional ELISA usually requires a minimal tear volume per analyte to be tested, which makes the techniques hardly applicable in the clinical routine setting. MULTIPLEX protein analysis allows simultaneous measurement of a panel of cytokines and to identify distinct cytokine profiles associated with DED in tears, requiring a smaller tear volume sample compared to using multiple ELISAs. Regrettably, there are few data comparing these methods in tears: the use of different tear analysis methods makes it difficult to compare data across studies, as it has been discussed for the great inconsistency in tear IL-6 levels determined with ELISA or MULTIPLEX techniques [27].

In the aqueous humor [28] a greater number of cytokines were detected and with increased sensitivity with MULTIPLEX than with ELISA techniques. However, when the number of analytes increases, the risk of non-specificity also increases, and the platforms should be compared with respect to accuracy, sensitivity and robustness of data provided.

As the cytokine levels can be influenced by many factors, the following issues might help improving the quality of data and drive the future research on tears of DED patients. As cytokine secretion follows a circadian rhythm, the time of sampling should be kept in consideration, during trials [29]. The cytokine levels can be influenced by medications, therefore a suspension should be observed before sampling [30]. In addition, the cytokine levels should be investigated with appropriate methods, to be selected on the level of sensitivity needed to measure a specific analyte, and, most importantly, the inter-laboratory quality assurance should be performed to standardize the method [30]. 

## 4. Methods

### 4.1. Search Strategy

This review was performed in accordance with the preferred reporting items for systematic reviews and meta-analyses (PRISMA) statement [31]. The PRISMA statement is a 27-item checklist which has a rational design for improving the quality of reporting systematic review and meta-analysis studies. Before the study is conducted, the authors (PV, MR, MP, GG) developed the study protocol. We searched PubMed, Embase, Web of Science, Ovid, Cochrane Database, Scopus databases for original articles published up to September 30, 2019, with no back date restriction, using the keywords (all languages) (“Cytokine*” OR “Chemokine*” OR “Interleukin*” OR “Human tear*” OR “biomarker*” OR “multiplex” OR “Luminex” OR “ELISA”) AND (“Dry eye” OR “Keratoconjunctivitis sicca”) without any limitation. In addition, references of selected retrieved articles were scanned manually to identify any additional studies. The searches were conducted by 2 independent investigators (MR, LT). Any discrepancies were resolved by discussion or by input from the third reviewer (PV).

### 4.2. Eligibility Criteria

The articles were considered eligible if the studies met the following inclusion criteria: (1) study type: case-control; (2) population: patients having DED of any etiology; (3) purpose: measurement of pro-inflammatory mediator concentrations in tears; (4) outcome variables for qualitative synthesis: to report the different concentrations of tear cytokines between non-DED control subjects and DED patients. Exclusion criteria were: (1) studies that employed sources other than those of interest (e.g., peripheral blood); (2) lack of a control group; (3) lack of data required for meta-analysis; (4) sorts of publications other than original articles (e.g., abstracts from conferences, letters, correspondence, reviews, duplicate publications, full texts without raw data available for retrievals).

### 4.3. Study Selection

After removing duplicate publications, two reviewers (PV, MR) independently screened the title and abstracts of all identified citations. The full text of citations judged as potentially eligible were obtained and independently screened for eligibility by two reviewers (PV, MR). Any disagreement in determination of the eligibility of each study was resolved by discussion with all authors.

### 4.4. Data Extraction

Two reviewers (MR, LT) independently extracted the following data from each included publication: first author name; year of publication; location; cytokine(s) measured; the assay technology; scales that were employed for cytokine measurement; tear sample collection; DED etiology; numbers of subjects; demographic characteristics (e.g., age and sex); tear cytokine levels (mean and standard deviation [SD]) in both the patient and the control groups. Studies with data reported in figures or with missing data were excluded in the quantitative synthesis. In case of discrepancies between the two reviewers, the manuscripts were revisited and agreed on by discussion.

### 4.5. Quality Assessment

As recommended by the Cochrane Collaboration [32], the quality of studies included in the between-group meta-analyses was assessed using the Newcastle–Ottawa Scale (NOS) designed for non-randomized studies [33]. This is formulated for the assessment of three main aspects of case-control studies: sample selection, comparability of cases and controls and outcome with a maximum of 8 stars. Each study was graded with A, B or C, based on the GRADE quality analysis criteria [34].

### 4.6. Data Analysis

Each cytokine was studied by means of meta-analytic methods and forest plot graphs. For each study the cytokine levels have been expressed as mean and SD for both DED and control groups. For each cytokine a figure with two graphs was presented: on the top a forest plot combined the mean values with 95% confidence intervals of control subjects in each study (referred as effect size, ES); on the bottom the standardized mean difference (SMD, referred as difference in means between the two groups) between the DED patients and control subjects mean values with 95% confidence intervals was reported. The heterogeneity across studies was calculated using the I^2^ index. If the same control group is used as comparison with multiple DED subgroups, the control values entered only once in the analysis of the heterogeneity.

The meta-analyses were performed with both the Inverse Variance method (fixed effect) and the DerSimonian–Laird method (random effect) [35]. The Cochrane guidelines state that an I^2^ value > 50% shows a high heterogeneity across studies (https://training.cochrane.org/handbook/current/chapter-10). To deal with inherent technical differences among studies, we conducted stratified metanalyses according to the MULTIPLEX-based or the enzyme-linked immunosorbent assay (ELISA)-based studies.

Statistical analyses were performed by using Stata version 14.2 SE (Stata Corp., College Station, TX, USA). A *p*-value < 0.05 was considered as statistically significant.

## 5. Conclusions

In conclusion, the major finding of the present review is that tear cytokine research in DED needs substantial improvement. The present meta-analysis was limited at the level of the literature search to cytokines, chemokines and interleukins; however, other inflammatory markers (e.g., complement proteins, metalloproteinases) may have also been informative, but quantitative data available are still few. There is also a need for defined and applied quality standards for study design and analytical performance and for standardization rules for study reports and manuscripts. Possible confounding factors of cytokine levels should be controlled, and a bigger sample size should be investigated.

## Figures and Tables

**Table 1:** Newcastle-Ottawa quality assessment scale for case control studies.

	Selection		Comparability		Outcome	
Study	Reviewer A	Reviewer B	Reviewer A	Reviewer B	Reviewer A	Reviewer B
Benitez del Castillo et al. [16] Cocho et al. [9] Landsend et al. [10] Lee et al. [11] Mrugacz et al. [12] Nair et al. [13] Solomon et al. [8] Tishler et al. [14] Yoon et al. [15] Zhao et al. [17] Acera et al. [18] Massingale et al. [19] Lam et al. [7]	★★★ ★★★★ ★★★ ★★★ ★★★★ ★★★ ★★★★ ★★★ ★★★★ ★★★ ★★★★ ★★ ★★★	★★★ ★★★★ ★★★ ★★★ ★★★★ ★★★ ★★★★ ★★★ ★★★★ ★★★ ★★★ ★★★ ★★★★	★ ★★ ★ ★ ★ ★ ★★ ★ ★ ★ ★ ★ ★	★ ★ ★★ ★ ★ ★ ★★ ★ ★ ★ ★ ★ ★	★★★ ★★★ ★★★ ★★★ ★★★ ★★★ ★★★ ★★★ ★★★ ★★★ ★★★ ★★ ★★★	★★★ ★★★ ★★★ ★★★ ★★★ ★★★ ★★★ ★★★ ★★★ ★★★ ★★★ ★★ ★★★

The scale is formulated for the assessment of three main aspects of case-control studies: sample selection, comparability of cases and controls and outcome with a maximum of 8 stars.

**Table 2:** Summary of data from the studies investigating tear inflammatory mediators in DED, included in the quantitative analysis.

Study	Study Group	Patients (*n*)	Tear Sample—Tear Collection	Cytokines Analysis	Results
Benitez-del-Castillo [16]	DED vs. control	30	Unstimulated tears—micropipette	Multiplex (Luminex R-200)	Higher levels of IL-1β, IL-2, IL-6, IL-8, IL-10, TNF-α and lower levels of VEGF in patients with DED
Cocho [9]	Chronic GVHD with DED vs. control	22	Unstimulated tears—capillary tube	Multiplex (Luminex IS-100)	Higher levels of IL-1Ra, IL-1β, IL-2, IL-6, IL-8, IL-10, IL-17A, and IFN-γ lower levels of EGF and IP-10 in patients with GVHD
Landsend [10]	DED w/aniridia vs. controls	35	Eluted from Schirmer strips—capillary tubes	Multiplex (Luminex IS 100)	A number of pro-inflammatory cytokines (IL-1β, IL-9, IL-17A, eotaxin, FGF2, and MIP-1a) are significantly elevated in tear fluid from DED w/aniridia patients, and correlate with parameters for MGD in aniridia.
Lee [11]	SS DED vs. non-SS DED vs control	49	Flush tears—micropipette	BDTM Cytometric Bead Array	Higher levels of IL-2, IL-4, IL-6, IL-10, IL-17A, and TNF-α in patients with SS-related DED
Mrugacz [12]	DED with depression vs. control	32	Unstimulated tears—micropipette	ELISA	Higher levels of IL-6, IL-17A and TNF-α in patients with DED and depression
Nair [13]	DED w/o GVHD vs. DED w/GVHD vs. control	32	Eluted from Schirmer strips—capillary tubes	Multiplex (Bio-plex-pro; Millipex)	Elevated levels of IL-2, IL 6, IL 8, IL 10, IL 12AP70, IL 17A, TNF-α, IFN-γ, and VEGF in DED w/GVHD eyes as compared to DED w/o GVHD and control eyes.
Solomon [8]	DED vs. control	40	Tears adsorbed with polyester wick	ELISA	Higher levels of IL-1α and IL-1β and lower levels of precursors IL-1β in patients with DED
Tishler [14]	pSS vs. control	24	Unstimulated tears—micropipette	ELISA	Higher levels of IL-6 in patients with primary SS
Yoon [15]	SS DED vs. non-SS DED vs. control	32	Unstimulated tears—micropipette	ELISA	Higher levels of IL-6 and TNF-α in patients with DED; higher levels of IL-6 in patients with SS-related DED
Acera [18]	DED vs. control	46	Tears adsorbed with cell sponge weck	ELISA	Higher levels of IL-1β and IL-6 in tear fluid of patients with DED and DED associated with blepharitis than in the control group
Lam [7]	DED vs. control	40	Unstimulated tears—micropipette	Multiplex (Luminex Beadlyte)	Higher levels of IL-1β, IL-6, IL-8, IL-12, and TNF-α in patients with DED
Zhao [17]	DED vs. control	70	Unstimulated tears—capillary tube	Multiplex (Luminex R-200)	Higher levels of TNF-α, IL-1α, IL-1β, IL-6, IL-8, IL-10, IL-12P70, IL-13, IFN-γ, and MIP-1a in DED patients compared with normal participants
Massingale [19]	DED vs. control	14	Unstimulated tears—microcapillaries	Multiplex (Luminex 100 TM)	Higher levels of IL-1β, IL-2, IL-4, IL-5, IL-6, IL-8, IL-10, IFN-γ, and TNF-α in DED patients

DED, dry eye disease; VEGF vascular-endothelial growth factor; EGF, epidermal growth factor; IP 10 interferon gamma-induced protein 10; ELISA, enzyme-linked immunosorbent assay; GVHD, graft-versus-host disease; IFN, interferon; IL, interleukin; MCP, monocyte chemoattractant protein; MGD, meibomian gland dysfunction; MIG, monokine induced by interferon-γ; MIP, macrophage inflammatory protein; POAG, primary open-angle glaucoma; SS, Sj*ö*gren syndrome; TNF, tumor necrosis factor; FGF fibroblast growth factors.

## Supplementary Materials

The following are available online at https://www.mdpi.com/1422-0067/21/9/3111/s1.
