# Supplementary material for: Dry Eye Disease and Tear Cytokine Levels—A Meta-Analysis
Source: Int J Mol Sci. 2020 Apr 28;21(9):3111. doi: 10.3390/ijms21093111 (PMC7246678; doi:10.3390/ijms21093111)
Supplement: Supplementary file 1 [file ijms-21-03111-s001.pdf]

Table 3. Summary of data from the meta-analysis, containing for each cytokine the calculation of heterogenenity among controls, the heterogeneity among DED, and the random effect meta-analyses, subdivided between Multiplex and ELISA techniques.

np=not present; SMD=Standardized Mean Difference; CI=Confidence interval.

	Heterogenity among controls					Heterogeneity among DED					Random effects meta-analysis				
	mean (pg/mL)	95%CI (pg/mL)	p	I2 (%)	p	mean (pg/mL)	95%CI (pg/mL)	p	I2 (%)	p	SMD	95%CI	p	I2 (%)	p
Overall	*18,68*	*10.15-27.21*	*0,0001*	*96,6*	*0,0001*	*31,54*	*23.93-39.15*	*0,0001*	*98,4*	*0,0001*	*0,61*	*0.20-1.03*	*0,004*	*78,3*	*0,0001*
Multiplex	10,06	2.43-17.69	0,01	95,5	0,0001	18,61	8.70-28.53	0,0001	92,5	0,0001	0,51	0.30-0.72	0,0001	0	0,773
Elisa	38,9	19.56-58.24	0,0001	86,2	0,007	44,27	37.72-50.83	0,0001	98,4	0,0001	1,02	-0.88-2.92	0,293	94,1	0,0001
Overall	*12,88*	*9.81-15.96*	*0,0001*	*99,7*	*0,0001*	*6,84*	*4.00-9.69*	*0,0001*	*96,7*	*0,0001*	*0,35*	*-0.18 - 0.87*	*0,197*	*77,5*	*0,0001*
Multiplex	12,88	9.81-15.96	0,0001	99,7	0,0001	6,84	4.00-9.69	0,0001	96,7	0,0001	0,35	-0.18 - 0.87	0,197	77,5	0,0001
Elisa	np	np	np	np	np	np	np	np	np	np	np	np	np	np	np
Overall	*12,04*	*8.34-15.75*	*0,0001*	*96,6*	*0,0001*	*98,82*	*46.75-150.89*	*0,0001*	*100,0*	*0,0001*	*1,57*	*0.99-2.15*	*0,0001*	*90,3*	*0,0001*
Multiplex	13,40	6.59-20.20	0,0001	96	0,0001	146,08	67.67-224.49	0,0001	100	0,0001	0,98	0.46-1.50	0,0001	84,6	0,0001
Elisa	13,12	7.94-18.29	0,0001	97,8	0,0001	35,30	18.67-51.94	0,0001	99,2	0,0001	2,78	1.58-3.97	0,0001	88,5	0,0001
Overall	*209,51*	*110.65-308.37*	*0,0001*	*98,5*	*0,0001*	*501,48*	*319.67-683.30*	*0,0001*	*97,0*	*0,0001*	*1,78*	*1.02-2.53*	*0,0001*	*88,7*	*0,0001*
Multiplex	209,51	110.65-308.37	0,0001	98,5	0,0001	501,48	319.67-683.30	0,0001	97	0,0001	1,78	1.02-2.53	0,0001	88,7	0,0001
Elisa	np	np	np	np	np	np	np	np	np	np	np	np	np	np	np
Overall	*4,51*	*2.22-6.81*	*0,0001*	*98,3*	*0,0001*	*7,87*	*3.23-12.52*	*0,001*	*96,9*	*0,0001*	*0,57*	*0.24-0.91*	*0,001*	*60,1*	*0,01*
Multiplex	4,51	2.22-6.81	0,0001	98,3	0,0001	7,87	3.23-12.52	0,001	96,9	0,0001	0,57	0.24-0.91	0,001	60,1	0,01
Elisa	np	np	np	np	np	np	np	np	np	np	np	np	np	np	np
Overall	*4,14*	*1.96-6.32*	*0,0001*	*85,9*	*0,0001*	*12,32*	*5.52-19.12*	*0,0001*	*96,1*	*0,0001*	*0,89*	*-0.02-1.80*	*0,054*	*93,2*	*0,0001*
Multiplex	5,75	1.38-10.12	0,01	86,9	0,0001	10,66	5.11-16.21	0,0001	88,1	0,0001	0,4	-0.13-0.93	0,14	78,2	0,001
Elisa	4,11	3.70-4.52	0,0001	/	/	18,48	16.46-20.50	/	/	/	3,52	2.74-4.29	/	/	/
Overall	*3,18*	*2.20-4.15*	*0,0001*	*99,2*	*0,0001*	*12,16*	*6.92-17.40*	*0,0001*	*99,3*	*0,0001*	*1,52*	*0.75-2.29*	*0,0001*	*92,8*	*0,0001*
Multiplex	9,38	5.66-13.10	0,0001	98,4	0,0001	11,94	7.53-16.34	0,0001	97,1	0,0001	0,76	0.36-1.16	0,0001	71,7	0,0001
Elisa	0,63	-0.59-1.86	0,311	99,8	0,0001	9,21	-1.57-19.99	0,094	99,4	0,0001	6,78	-3.83-17.39	0,211	98,8	0,0001
Overall	*118,79*	*19.10-218.49*	*0,02*	*98,3*	*0,0001*	*183,50*	*104.18-262.82*	*0,0001*	*97,2*	*0,0001*	*0,80*	*0.43-1.17*	*0,0001*	*31,2*	*0,201*
Multiplex	118,79	19.10-218.49	0,02	98,3	0,0001	183,50	104.18-262.82	0,0001	97,2	0,0001	0,80	0.43-1.17	0,0001	31,2	0,201
Elisa	np	np	np	np	np	np	np	np	np	np	np	np	np	np	np
